# An Internal Carotid Artery Dissection Masquerading as Optic Neuritis: A Case Report

**DOI:** 10.7759/cureus.12810

**Published:** 2021-01-20

**Authors:** Krishin Nandwani, Shin Ying Thng, Poongkulali Anaikatti

**Affiliations:** 1 Emergency Medicine, Changi General Hospital, Singapore, SGP; 2 Accident and Emergency, Changi General Hospital, Singapore, SGP

**Keywords:** dissection, internal carotid artery (ica), optic neuritis, stroke, arterial emboli

## Abstract

Carotid artery dissection is an important cause of stroke, especially in the young. We present a 43-year-old lady, with a known background of headaches, who was referred to the Emergency Department with a headache, dilated pupil, and acute monocular blurring of vision. She was later found to have an internal carotid artery dissection (ICAD) with diffuse ipsilateral hemispheric involvement after being initially managed for atypical optic neuritis. This case report aims to provide further insight into an atypical presentation of a carotid artery dissection, with the intent of assisting the clinician in identifying such cases during the initial presentation.

## Introduction

Despite accounting for approximately 15% of strokes in young adults [1], making a diagnosis of a cervicocephalic dissection at initial presentation has a reputation of being notoriously difficult. Patients generally present with a wide range of symptoms that may masquerade the underlying pathology. The symptomology and pathophysiology of internal carotid artery dissection (ICAD) were first intensively studied in the 1970s by Fischer et al. [2] and by 1985, Caplan et al. [3] described ICAD to cause prominent neck and facial pain, headache, ipsilateral Horner's syndrome, episodes of ipsilateral cerebral hemisphere and retinal ischemia. and sometimes "migrainous" visual accompaniments. These studies have laid down the foundations of the currently commonly known ipsilateral ICAD triad of unilateral headache, ipsilateral Horner’s syndrome, and ischaemic symptoms. However, less than a third of patients present with the classic triad [4]. Headaches are the most common of the triad, being reported in 68% of ICAD cases, and are non-specific in character with pain ranging from mild to severe [5]. This case report aims to present an atypical presentation of an ICAD and discuss the pathophysiology leading up to her initial misdiagnosis.

## Case presentation

A 43-year-old Indian female with a history of hypothyroidism and hypertension was referred to the Emergency Department in the evening for a two-day history of headaches (of waning severity) and blurring of vision. Her headache started the night prior and she described it to be typical of her usual migraines which she has experienced for five years. The headache was tolerable and localised to the vertex of her head, associated with nausea, vomiting, photophobia, and phonophobia. There were no visual, speech, or focal neurological impairments at the time. Furthermore, there was no described neck pain and she was able to sleep after taking oral analgesia. Upon waking up in the morning, her headache had persisted and was progressively becoming more severe, which was unusual for her typical migraines. She tolerated this pain and by early afternoon took more oral analgesia before proceeding to sleep again. When she awoke, she noted the vision in her right eye to be blurred and more dim compared to her left eye, but there was no ocular pain. At this time, she experienced momentary weakness in her left upper limb and left lower limb which resolved spontaneously within a few minutes. She went to her General Practitioner who prescribed her cafergot and domperidone prior to referring her to the Emergency Department for evaluation of her headache with right eye blurring of vision.

Upon arrival at the Emergency Department, her headache was still present but had improved with medications. At initial examination, she was not in distress and was walking independently. Her Glasgow Coma Scale (GCS) was 15/15 and her vital signs were within normal range. Her physical examination was remarkable for a right eye mydriasis with visual acuity (VA) of 6/36. Her right eye was minimally reactive to light with a grade 3 relative afferent pupillary defect (RAPD). The extraocular movements were full and there was no nystagmus. The rest of her cranial nerves and neurological examination showed no gross abnormality as well. There was no focal facial tenderness. 

Investigations

An urgent non-contrast computed tomography (CT) brain scan did not reveal any acute abnormalities. Blood tests were only significant for an erythrocyte sedimentation rate (ESR) of 27. The full blood count and renal panel were grossly unremarkable.

She was reviewed by an ophthalmologist in the emergency room, and further examination of her right eye demonstrated that the patient’s visual field was only limited to the supero-nasal region. Her optic disc was hyperaemic and swollen (worse infero-nasally) with blurred margins, normal peripheral retina and vessels, and a normal cup-to-disc ratio. There was no cherry red spot.

Impression and disposition

The initial impression was that of atypical optic neuritis with an intent to rule out either a compressive or non-arteritic anterior ischemic optic neuropathy (NAION). She was admitted under the department of ophthalmology with a plan for an early magnetic resonance imaging (MRI) of her brain and anterior visual pathway.

Progress in the ward

Upon review the following morning, the patient complained of a complete loss of vision to her right eye which she noted upon waking up in the middle of the night. This was associated with some pressure sensation in the said eye with no further neurological complaints. She was only able to perceive light in all four quadrants of her right eye with projection limited to the right temporal quadrant. Her RAPD had not improved and her optic disc was pink and swollen. The right macula was flat with a good foveal reflex. Goldmann Visual Fields (GVF) of her right eye demonstrated a small residual temporal island. The overall examination findings were suggestive of a right atypical optic neuritis suspicious of a neuromyelitis optica spectrum disorder.

**Figure 1:**
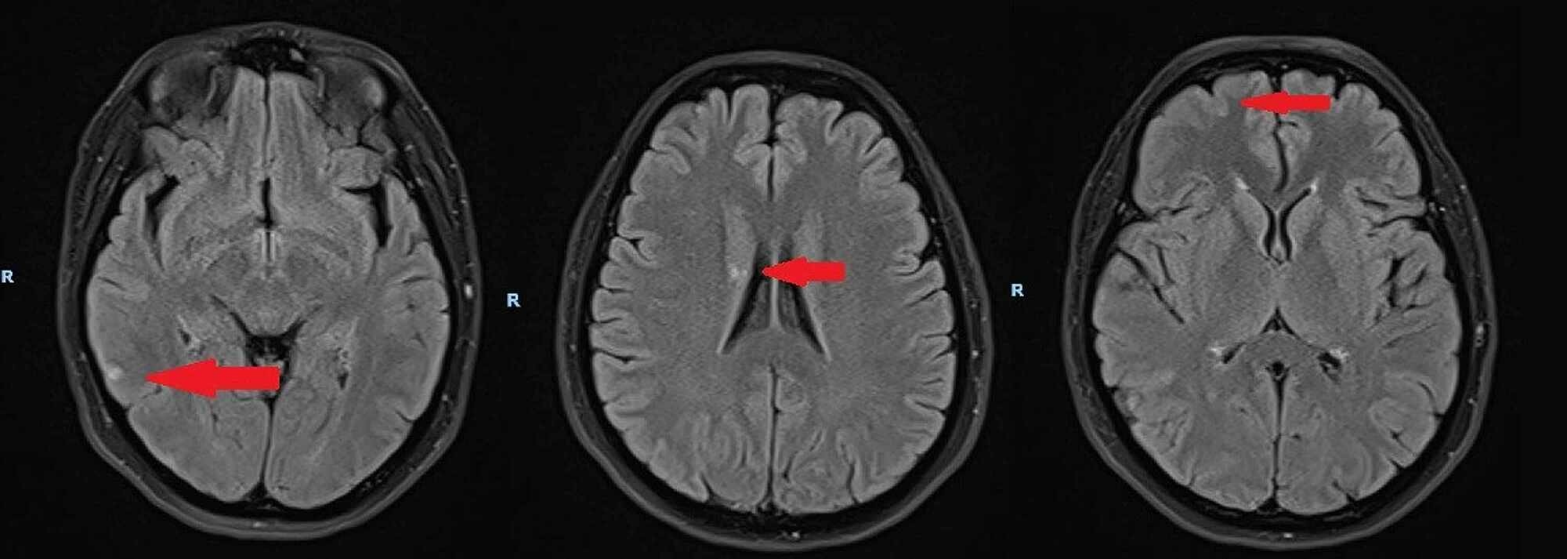
MRI Brain demonstrating areas of acute non-haemorrhagic infarcts

An MRI of the brain (Figure 1) and orbits done that day demonstrated scattered acute non-haemorrhagic infarcts. This was observed in the right frontal, parietal, occipital, and temporal lobes in the cortices, subcortical white matter, and corona radiata. There was loss of right internal carotid artery (ICA) flow void with suggestion of a double lumen proximally. The right optic disc and optic nerve appeared unremarkable.

**Figure 2:**
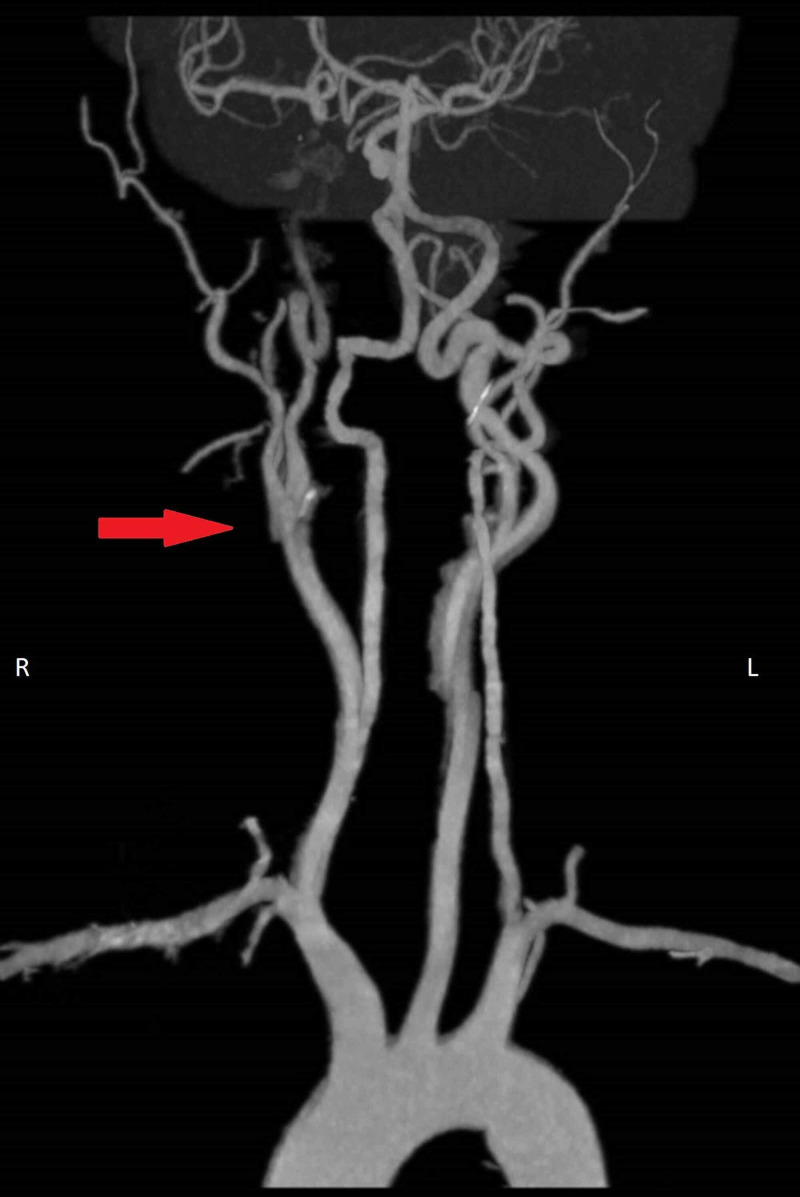
CT angiogram demonstrating severe attenuation of right internal carotid artery (ICA) just beyond take-off and poor opacification up to cavernous segment. Motion artefacts degrade quality of picture.

An urgent CT angiogram (Figure 2) demonstrated severe attenuation of almost the entire right ICA with narrowing just beyond its take-off and poor opacification up to the cavernous segment with suggestion of a double-lumen/intimal flap from the level of C2.

The patient was immediately admitted to the stroke unit and her ICAD was managed conservatively with anti-platelets. Her right eye vision did not improve significantly during her stay in the hospital despite intravenous acetazolamide and topical brimonidine tartrate to reduce intra-orbital pressure and improve perfusion in the eye. She developed no other neurological complaints.

## Discussion

Carotid artery dissections occur when there is a break in the lining of the artery leading to a collection between the layers of the arterial wall known as an intramural haematoma. They have an incidence of 2.6 per 100,000 per year [6] and make up 20% of strokes occurring in patients below 45 years of age [7]. ICAD can occur in any patient regardless of age or cardiovascular risk factors [8]. Their aetiology is largely unknown; however, known precipitating factors typically include a recent neck trauma (of any severity including coughing or yoga stretching) [9] and patients with hereditary connective tissue diseases such as Ehler-Danlos syndrome, Marfan syndrome, or alpha-1-antitrypsin deficiency are at higher risk of developing ICAD [8]. Our patient had no history of connective tissue disorders but later during her stay had revealed participation in an online Zumba dance class the night prior to her presentation, which could have been a precipitating factor. Other factors that can affect the integrity of the arterial lining include recent infections, smoking, hypertension, and contraceptive use [8]. Of note, ICADs have also been reported with migraines [10].

As previously elaborated, the presentation of ICAD is hardly textbook. The headache being the most common of the ICAD triad has also been shown to respond well to analgesics like sumatriptan [11] thereby making it even harder to diagnose. The monocular drop in visual acuity from ICAD can range from transient to permanent and results from ischaemic optic neuropathy (ION), central retinal artery occlusion (CRAO)/branch retinal artery occlusion (BRAO), or ophthalmic artery occlusion (OIS) [12].

ION is one of the commonest causes of blindness in people over the age of 50 [13] and this occurs due to infarction of the optic nerve. The site of ischaemic damage is used in the classification of ION as fundoscopic examination helps to delineate the site involved - anterior ischaemic optic neuropathy (AION) if the initial fundic examination shows disc oedema, and posterior (retrobulbar) ischaemic optic neuropathy (PION) if there is no initial disc oedema but subsequent examination exhibits optic disc atrophy [14]. Giant cell arthritis and nonarterial ION make up 90% of the causes of ION while only 4% of ION occurs due to ICAD [12,15]. CRAO/BRAO can result from an occlusion or decrease in perfusion of the central retinal artery. A cherry red spot with signs of severe retinal ischaemia is pathognomonic for CRAO while retinal whitening with associated oedema along a branch of the retinal artery is suggestive of BRAO. In the setting of ICAD, it is rare to get emboli in the ophthalmic artery as there is a reversal of blood flow within the artery to compensate for a reduced blood flow to the ipsilateral part of the brain [16]. OIS occurs due to hypoperfusion secondary to occlusion of the ophthalmic artery and is also rare. Our patient was diagnosed with a right ophthalmic artery occlusion secondary to the ICAD.

Horner syndrome is characterised by ipsilateral ptsosis, anhidrosis, and miosis (constriction of the pupils). These occur due to interruption of the sympathetic chain secondary to the compressive effects of the intramural haematoma resulting from ICAD. A painful Horner syndrome with emphasis on headache and neurological signs or complaints cautions the attending doctor to consider ICAD high in the differential. Our patient had no signs of Horner’s syndrome - in fact, she presented with a dilated pupil. A dilated pupil in the setting of a carotid artery dissection was first described in 1998 as a case report and was postulated to occur due to cerebral ischaemia [17]. Ipsilateral mydriasis can also occur in Pourfour du Petit syndrome (PDPS), a rare entity that is commonly described to be the opposite of Horner syndrome as it is also accompanied by hyperhidrosis and eyelid retraction. PDPS occurs due to hyperactivity of the sympathetic chain and was suggested to be an early sign of ICAD in 2019 [18].

## Conclusions

In conclusion, ICAD remains a diagnostic challenge. However, an improved understanding of its varied presentations will lead to increased chances of its detection by the attending clinician. There are five main learning points from this case report - the ICAD triad is not a reliable on-the-ground tool for the diagnosis of ICAD, the headache in ICAD can be of mixed severity, respond well to oral analgesics and does not necessarily have to be ipsilateral, a dilated pupil is just as significant as a constricted pupil as either Pourfour du Petit syndrome or Horner’s syndrome can be the initial complaint of an ICAD, and ophthalmic artery emboli are rare in ICAD due to a reversal of blood flow within the artery to compensate for brain ischaemia.
